# Case of a Wound Penetrating the Cavity of the Abdomen

**Published:** 1800

**Authors:** Henry Fryer

**Affiliations:** Surgeon at Stamford.


### XII. *Case of a Wound penetrating the Cavity of the Abdomen. By the same.*

SUNDAY, Feb. 4, 1798, I was ſent for to the daughter of —— Roadley, of Clipſham in Rutland, on the following account:— She had been thrown down in a farmer's yard, and gored by a cow. The accident had happened ſix hours before I ſaw her. I found a very large quantity of the inteſtines had paſſed from the abdomen; but, at firſt, I could not proceed in my examination, ſo great an adheſion had taken place between the inteſtines and a ſilk handkerchief which had imprudently been laid to them. Having, with very conſiderable difficulty, removed this, by ſoaking it well with warm water, the inteſtines were diſcovered prodigiouſly inflated, and with ſome appearance of inflammation, owing to the ſmallneſs of the opening made by the cow's horn. It was ſome time before I was able to introduce my finger into the orifice, and upon it a probe pointed knife, with which I dilated lated the wound fufficiently to fuffer a ftill larger quantity of the inteftines to pafs out: this immediately reduced the fize of them, fo that without much trouble I was enabled to return the whole into the cavity of the abdomen, and then had an opportunity (which I had not before from the number of folds that lay out) of examining more minutely at what part the wound had been made. This I found to be juft above Poupart's ligament; and I alfo difcovered that I had wounded the epigaftric artery. With fome difficulty I fecured this veffel; and the wound was then drawn together by two ftitches, and flips of adhefive plafter, and over thefe comprefs and bandage. I gave the patient fifty drops of laudanum; and directed her to take fome caftor oil the next morning. She continued very ill the whole of the next day, with a low trembling pulfe, great weaknefs and pallid countenance, but without much pain. The oil was repeated, and two clyfters given, which on Tuefday morning produced fufficient evacuations. From this time fhe had no bad fymptom; the wound digefted kindly: fhe was kept entirely on nourifhing liquid diet; the ftate of the bowels was particularly attended to; an opiate was given at night; and after ſome time, ſhe took decoction of bark.

In ſix weeks the wound was healed, and ſhe gradually recovered her ſtrength. She was about ſixteen years old.

*Stamford, June* 1, 1798.

